# A Comparison Between Piezosurgery and Conventional Osteotomies in Rhinoplasty on Post-Operative Oedema and Ecchymosis: A Systematic Review

**DOI:** 10.1007/s00266-022-03100-5

**Published:** 2022-09-26

**Authors:** Janneta Kisel, Manaf Khatib, Naveen Cavale

**Affiliations:** 1 Faculty of Life Sciences and Medicine, King’s College London, Guy’s Campus, Great Maze Pond, London, SE1 1UL UK; 2 Lister Hospital, East and North Hertfordshire NHS Trust, Stevenage, UK; 3 King’s College Hospital and Guy’s & St.Thomas’ Hospitals, King’s College Hospital and Guy’s & St.Thomas’ Hospitals NHS Trusts, London, UK

**Keywords:** Rhinoplasty, Oedema, Ecchymosis, Osteotomy, Piezosurgery

## Abstract

**Abstract:**

Piezosurgery use has become increasingly prevalent in osteotomies. Piezoelectric ultrasound waves can cut bone effectively, and some studies have shown reduced post-operative morbidities compared to conventional osteotomies. Oedema and ecchymosis are common complications of rhinoplasty and can impact patient satisfaction, wound healing, and recovery. We aim to provide an up-to-date comparison of post-operative oedema and ecchymosis in piezosurgery and conventional osteotomies. A literature search was conducted using the following online libraries; Pubmed, Cochrane, Science Direct, and ISRCTN (International Standard Randomised Controlled Trial Number). English publications between 2015 and 2020 were included. A systematic review was completed, and a comparison of oedema and ecchymosis in piezosurgery and conventional osteotomies was examined alongside other outcomes such as pain, mucosal injury, and surgery time. Eight randomised controlled trials (RCTs) met our criteria with a combined total of 440 patients: 191 male and 249 female. Piezosurgery had statistically significant (*p *< 0.05) reduction in short-term oedema compared to conventional osteotomies in 75% of the papers included, and in 50% this persisted across the whole follow-up period. Similarly, ecchymosis scoring was initially statistically lower (*p *< 0.05) in piezosurgery in 87.5% of the RCTs, and in 75% this persisted across the whole follow-up period. A reduction in pain (*p *< 0.05) and mucosal injury (*p *< 0.05) was also seen in piezoelectric osteotomies. The length of surgery time varied. Piezoelectric osteotomies reduce oedema and ecchymosis compared to conventional osteotomies, in addition to improving pain and mucosal injury. However, disadvantages such as length of surgery time and cost have been reported.

**Level of Evidence III:**

This journal requires that authors assign a level of evidence to each article. For a full description of these Evidence-Based Medicine ratings, please refer to the Table of Contents or the online Instructions to Authors  www.springer.com/00266.

## Introduction

Rhinoplasties were the fourth most common cosmetic surgical procedure performed worldwide in 2020 [1], predominantly performed to improve aesthetic appearance or function of the nose. A rhinoplasty can involve external or internal incisions, alteration of the nasal turbinates and valves, tip reduction, reshaping of the dorsal hump, and symmetrical reshaping of the nasal vault via an osteotomy. Rhinoplasties can be performed open, where the nasal skin and subcutaneous tissue is dissected to expose the cartilage, or in a closed manner.

Conventionally, osteotomies are performed in rhinoplasty to create an optimal bony vault preventing functional airway obstruction [2]. This is performed using mechanical energy with a 2mm osteotome to cut through the nasal bone in order to mobilise the bony support structure of the nose, allowing space for final alterations and correction of deformities [3]. There is still debate about the optimal approach for an osteotomy as multiple approaches can be taken including internal, external, perforating, and cutaneous [4]. Additionally, osteotomies can be performed at any stage of a rhinoplasty. This mechanical force can lead to irregularities in bone cutting and trauma to the surrounding mucosa resulting in oedema and ecchymosis [5].

Piezosurgery was first applied in other oral and maxillofacial surgeries by Tomaso Vercellotti in 1988 [6]. It was then first used in rhinoplasty by Dr Massimo Robiony in 2004 [7] and uses ultrasonic vibrations to cut bone. An electric current is passed across two piezoelectric crystals, generating oscillations of a frequency usually between 25 and 30Hz which are amplified and converted to the scalpel. The vibrating Piezosurgery Medical Device (PMD) scalpel is moved continuously across the ideal osteotomy line to generate a cut through various tips [8], denaturing proteins and emulsifying the bone which is subsequently removed by continuous irrigation or suction. This prevents mechanical or thermal energy damage to the surrounding tissue whilst creating a precise fracture line [8, 9]. Whilst piezosurgery has been suggested to reduce oedema and ecchymosis, it may not prevent it completely. However, studies have shown higher costs and operating times in piezosurgery [10].

As the nose is a highly vascularised zone, oedema and ecchymosis are both common complications of rhinoplasties, often resulting in sub-optimal outcomes [11]. Oedema results from trauma during surgery, leading to water retention in the periorbital region, and has been shown to cause a reduction in visual acuity and healing outcomes. Ecchymosis results from breaking of blood vessels, creating bruising, impacting patient satisfaction, and healing. Some studies have shown piezosurgery leads to reduced oedema and ecchymosis due to the reduction in soft tissue damage [8]. Other complications such as necrosis and wound healing [12, 13] are not covered in the scope of this review.

Multiple studies have been carried out comparing the outcomes in conventional osteotomies and piezosurgery, although new literature is continuously produced. Therefore, we performed this systematic review of articles published between 2015 and 2020 to provide up-to-date information on the difference between conventional osteotomy and piezosurgery on oedema and ecchymosis, which may alter patient outcomes and satisfaction.

## Methods

### Search Strategy

A computer-based literary search was completed between December 2021 and January 2022 using the following bibliographic databases: Pubmed, Cochrane, Science Direct, and ISRCTN (International Standard Randomised Controlled Trial Number). No time restriction was initially applied, and the search criteria were as follows: “Rhinoplasty” AND (“Edema” OR “Ecchymosis”).

The inclusion criteria were: availability of the full text of the article, publication on or after January 2000; the article was a comparison between piezo surgery and osteotomies, a focus on the outcome variable being oedema or ecchymosis (or both), and living human studies only.

The exclusion criteria were: a predominant focus on septoplasty or septorhinoplasty, papers published before January 2000, papers not in English, no comparison between piezosurgery and osteotomies, no data on oedema or ecchymosis, cadaveric studies, and meta-analyses/literature reviews.

### Data Extraction/outcomes

All data were extracted independently. Following the application of the inclusion and exclusion criteria, 8 randomised controlled trials (RCTs) were obtained. The full text of these articles was analysed, and the following pieces of general information were collected about each study: first author, year of publication, mean age of participants, exclusion criteria for the study, type of study, distribution of participants between surgery types, open or closed, osteotomy technique, and post-operative interventions.

The following pieces of data were collected to assess outcomes: the time period of data collection, method of scoring oedema and ecchymosis, main findings regarding oedema and ecchymosis, and any other significant results. Where present, comparisons in the extent of decrease in oedema and ecchymosis over the follow-up period were recorded. It was assumed all patients received the same level of care.

In order to compare the level of oedema and ecchymosis between surgical techniques, data were recorded intermittently up to 7 days post-surgery.

Oedema and ecchymosis were recorded as follows:

- Oedema: 5 papers assessed eyelid oedema using the Kara and Gökalan (Adopted by Yucel) visual scale explained below [14], although two studies used a scale of 0-3, and one used 0-2.
  o Grade 1: Iris not covered by eyelid
  o Grade 2: Part of the iris covered by the eyelid
  o Grade 3: Full iris coverage with a flap of the eyelid
  o Grade 4: Complete closure and swelling of the eye
- Ecchymosis: 3 papers assessed eyelid ecchymosis using the Kara and Gökalan (Adopted by Yucel) visual scale explained below [14], although four studies used a scale of 0–4, and one used 0–2.
  o Grade 1: One third of the inner lower or upper eyelid affected
  o Grade 2: Two thirds of the inner lower or upper eyelid affected
  o Grade 3: Entire upper or lower eyelid effected

Some studies recorded post-operative pain with the visual analogue scale (VAS) scoring [15].

In all studies, piezosurgery and conventional osteotomies were performed on separate patients.

Other scoring systems have also been used. The sinonasal outcome test-22 (SNOT-22) evaluates nasal symptoms [16], peak nasal flowmetry (PNIF) measures air-flow through the nose, and the Connecticut Chemosensory Clinical Research Centre test (CCCRC) identifies odour detection [17].

### Quality and Bias Assessment

**Fig. 1:**
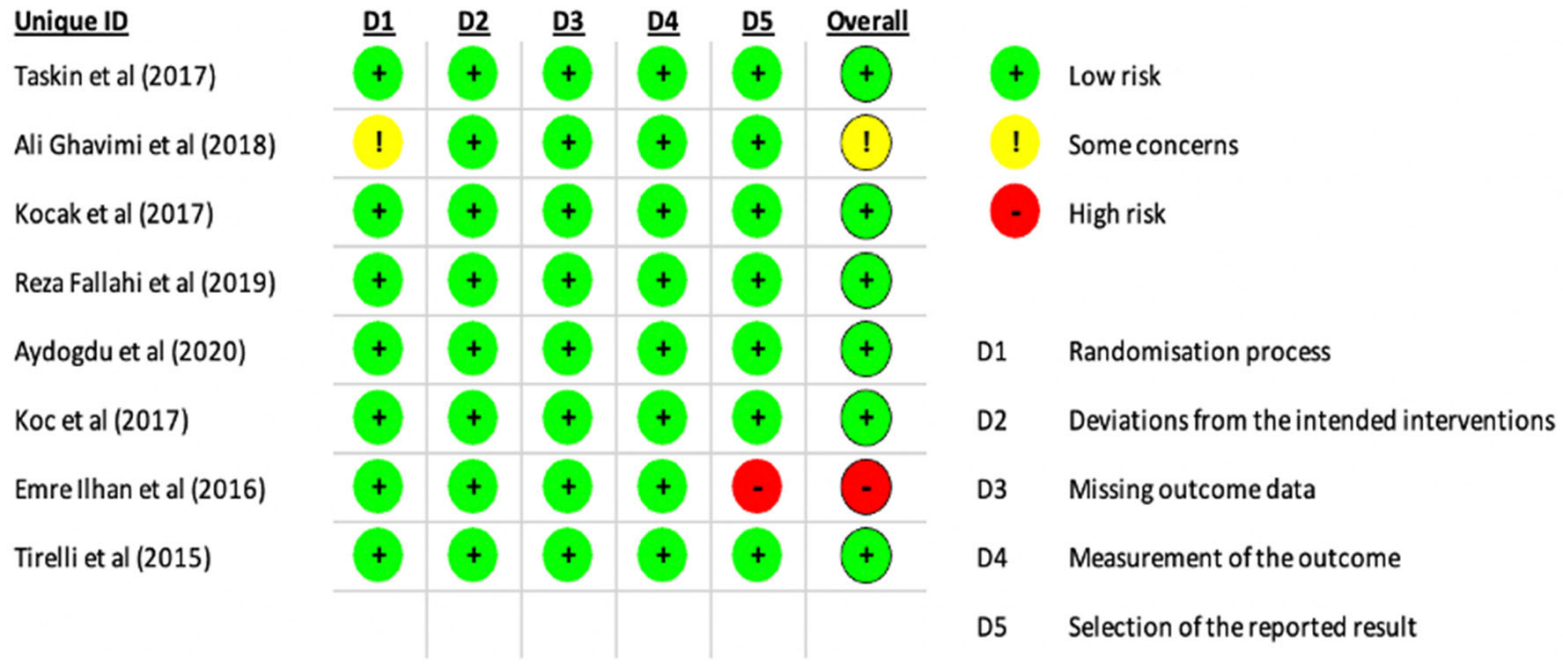
A risk of bias diagram for the included studies conducted using the Cochrane risk of bias assessment tool

This systematic review was performed according to recommendations from the Preferred Reporting Items for Systematic Reviews and Meta-analyses (PRISMA) statement [18]. One researcher assessed the quality of evidence using the Cochrane risk of bias assessment tool for randomised controlled trials to determine bias in the randomisation process, deviations from intended interventions, missing outcome data, measurement of the outcome, and selection of the reported data (Fig. 1). The majority of studies do not provide any bias concerns; however, one posed an intermediate risk of bias and one a high risk.

## Results

### Study Characteristics

**Fig. 2:**
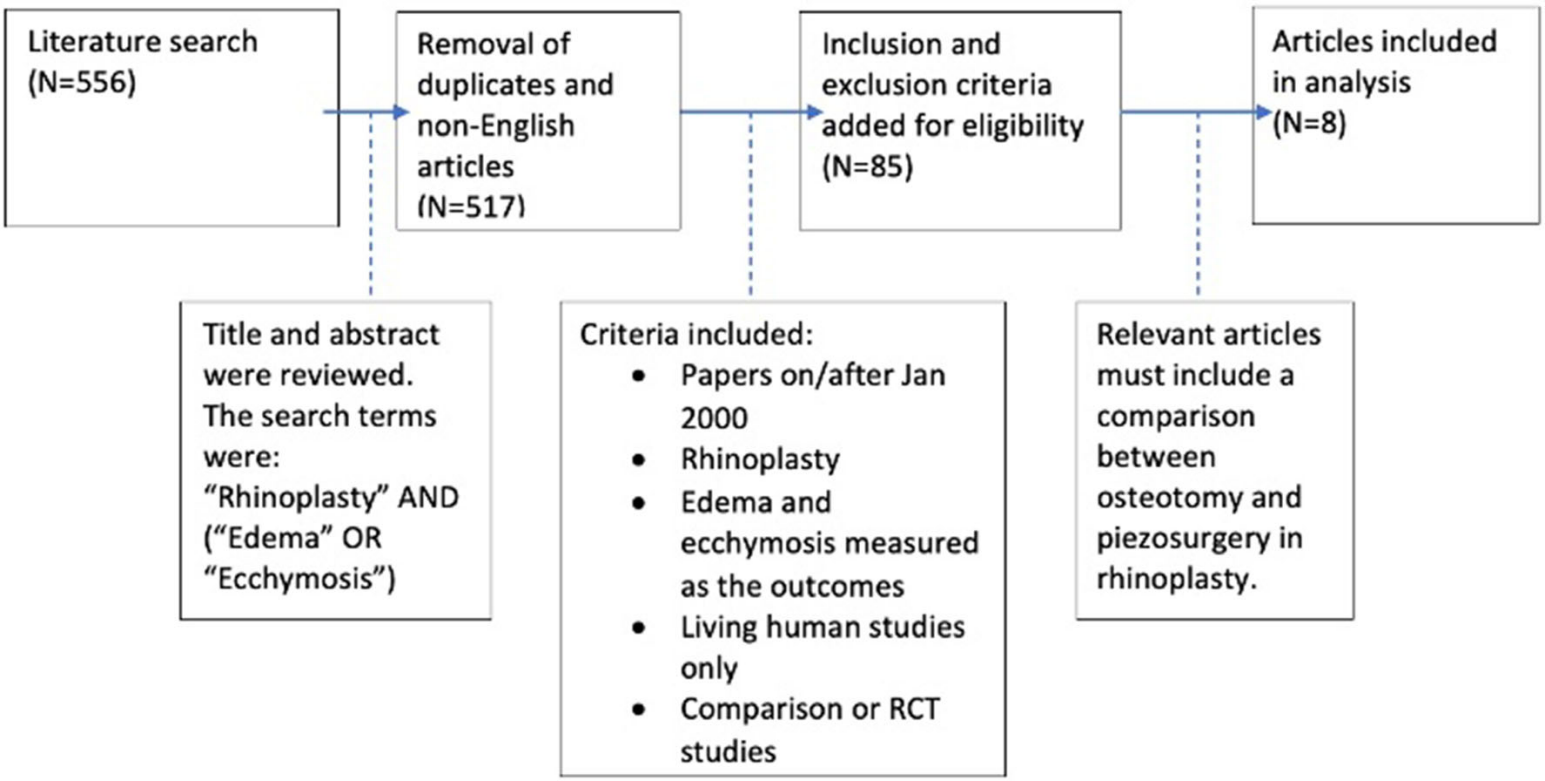
A flow diagram showing the literature search and study selection for this systematic review

On application of the search criteria, 556 articles were found. After reviewing and removing irrelevant abstracts, duplicates, and meta-analyses/systematic reviews, 85 papers remained. Further analyses of the articles resulted in 8 articles that fit the full criteria and were assessed in this review (Fig. 2). The publication years of the included articles ranged from 2015 to 2020. All 8 studies were RCTs.

### Patients

The articles compared the impact of piezosurgery and conventional osteotomies on post-surgical oedema and ecchymosis. Across the 8 studies, 440 patients underwent rhinoplasties; 191 (43%) males and 249 (57%) females. The mean age of participants ranged from 23.6 years to 28.5 years (Table 1). There was no control; patients either underwent conventional osteotomies or piezosurgery.

**Table 1:** Characteristics of the studies included in the systematic review

Author (year)	Number of patients	Male: Female	Mean age (years)	Exclusion criteria	Distribution of surgery	Open/ closed	Surgical process	Post-operative interventions
Taşkın et al. [19]	90	56 male:34 female	25.6	Patients with previous nasal surgery, use of anticoagulant drugs, hypertension, bleeding diathesis with chronic disease, chronic skin allergy, or inflammatory skin disease	Piezosurgery: 45 Conventional osteotomy: 45	Open	An median oblique and lateral osteotomy was performed using the selected instrument.	Cold ice packs were applied for 24h.
Ghavimi et al. [20]	66	33 female:33 male	N/A	Patients who were current smokers, patients with chronic rhinosinusitis, chronic diseases of the skin or rheumatology, nasal polyps, asthma, allergic rhinitis, patients with prior septoplasty or nasal beautification surgeries, ecchymosis or oedema before surgery for any reason, patients with thick skin or with a lateral bone thicker than 3mm, and patients who did not come back for post-operative examinations	Piezosurgery: 33 Conventional osteotomy: 33	Open	An intranasal lateral osteotomy was performed using the selected instrument.	For the first 24h all patients tilted their head upwards 30 degrees and a cold compress was applied for 15 minutes every hour. Patients were prescribed Cephalexin (500mg) every 8h for 5 days, Gelofen (400mg) every 6h for 5 days, and dexamethasone (8mg) every 8h for 24h.
Koçak et al. [21]	49	32 female:17 male	Piezosurgery:28.5Conventional osteotomy: 25.7	Patients with a history of rhinoplasty, an extremely wide nasal roof, a need for double lateral osteotomy, a narrow nasal roof and coagulopathy, smoking, and systemic diseases.	Piezosurgery: 24 Conventional osteotomy: 25	Open	An intranasal lateral osteotomy was performed using the selected instrument.	All patients were prescribed post-operative antibiotics BDS for 7 days, and acetaminophen BDS for 5 days. For the first 24h all patients tilted their head upwards 30 degrees and a cold compress was applied for 20 minutes every hour.
Fallahi et al. [22]	20	12 female: 8 male	Piezosurgery:24.9Conventional osteotomy: 24.0	Patients who were pregnant, used antidepressants, appeared noncompliant, or were unwilling to participate in the study	Piezosurgery: 10 Conventional osteotomy: 10	Open	An intranasal lateral osteotomy was performed using the selected instrument.	All patients were prescribed cephalexin (500mg every 6h), and acetaminophen (325mg) and codeine (10mg) for 7 days.
Aydoğdu et al. [23]	72	42 female:30 male	28.1	Patients who had undergone nasal surgery, patients with anticoagulant drug use, patients with bleeding diathesis, patients with chronic liver and kidney disease, patients with hypertension, patients with active rhinitis, and smokers	Piezosurgery: 36 Conventional osteotomy: 36	Open	Lateral osteotomy was performed with an external approach using the selected instrument.	All patients remained in a semi-seated position and a cold compress was applied for 15 minutes every hour during the first 24h. Oral analgesia and antibiotics were given for 7 days.
Koc et al. [24]	65	36 female:29 male	23.6	Patients with a history of smoking, presence of systemic diseases (such as cardiac disease, diabetes mellitus, hypertension, bronchial asthma, neurologic diseases), and use of any medications	Piezosurgery: 33 Conventional osteotomy: 32	Open	Lateral osteotomy was performed with a nasal approach using the selected instrument.	All patients remained in a semi-seated position, cold compresses were applied to the eyes and continually changed for 24h.
Tirelli et al. [25]	22	10 male:12 female	N/A	Patients who had already undergone a previous rhinoplasty procedure, or who presented a narrow nasal dorsum and a minimal hump	Piezosurgery: 10 Conventional osteotomy: 12	Closed	Lateral osteotomy was performed with an external percutaneous approach using the selected instrument.	6 days of antibiotic therapy, painkillers for 4 days (IV Acetaminophen 1 g in associ-ation with Tramadol 100 mg diluted in 100 ml saline solution TDS).
Ilhan et al. [26]	56	48 female:8 male	26.07	Patients who were current smokers; patients with chronic rhinosinusitis, chronic dermatologic or rheumatologic diseases, nasal polyposis, asthma, or allergic rhinitis; and patients who had previously undergone septoplasty or rhinoplasty	Piezosurgery: 34 Conventional osteotomy: 22	Open	Lateral osteotomy was performed with an internal approach using the selected instrument.	Dexamethasone (8g), prophylactic antibiotics (7 days), advice to apply a cold compress for 15 minutes every hour during the first 24h.

### Surgery Process

Open rhinoplasty was performed in 7 out of the 8 studies. All operations performed involved variations in lateral osteotomies compared to piezosurgery (Table 1). Most studies excluded patients who had previously undergone rhinoplasty or septoplasty. Common post-operative interventions used were cold compresses, antibiotics, analgesia, dexamethasone, and a semi-seated position. No drains were inserted post-surgery.

### Oedema and Ecchymosis Outcome Measurements

The trials used a variation in a scoring systems for both oedema and ecchymosis (Table 2).

**Table 2:** Scoring and findings of oedema, ecchymosis, and other complications in the studies included

Author	Days recorded	How oedema was recorded	Oedema results	How ecchymosis was recorded	Ecchymosis results	Other findings
Taşkın et al. [19]	Day 2 and 7	Scale of 0–4	No significant difference between the groups on both days. Significantly higher oedema on day 2 compared to day 7 in both groups (*p *< 0.05)	Scale of 0–4	No significant difference between the groups on both days. Significantly higher ecchymosis on day 2 compared to day 7 in both groups (*p *< 0.05)	N/A
Ghavimi et al. [20]	Day 3 and 7	Scale of 0–4	Higher oedema in conventional osteotomy than piezosurgery after 3 and 7 days (*p *< 0.001 and *p *= 0.049).	Scale of 0–3	Higher ecchymosis in conventional osteotomy than piezosurgery after 3, but not after 7 days (*p *> 0.117).	N/A
Koçak et al [21]	Day 2, 4 and 7	Scale of 0–4	Higher oedema in conventional osteotomy than piezosurgery after day 2 (*p *= 0.006), no significant difference on day 4 and 7. Conventional osteotomy group oedema decreased from day 2 to 7 (*p *< 0.001) Piezosurgery group oedema increased from day 2 to 4 (*p *= 0.007) but decreased day 4 to 7 (*p *< 0.001)	Scale of 0–4	Higher ecchymosis in conventional osteotomy than piezosurgery after day 2 and 4 (*p *< 0.001) and day 7 (*p *= 0.011). Significant decrease in ecchymosis for both groups from day 2 to 7 (*p *< 0.001 for both)	Conventional osteotomies took less time than piezosurgery (*p *< 0.001); however, the overall surgery duration was not significantly different. Pain on day 2 was significantly less in piezosurgery patients (*p *= 0.03). Mucosal injury was significantly less common in piezosurgery patients (*p *= 0.001).
Fallahi et al. [22]	Day 2 and 7	Scale of 0–4	Higher oedema in conventional osteotomy than piezosurgery after day 2 (*p *= 0.043), no significant difference on day 7 (*p *= 0.28)	Scale of 0–3	Higher ecchymosis conventional osteotomy than piezosurgery after 2 and 7 (*p *< 0.05).	VAS scoring showed reduced post-operative pain at every time point in the piezosurgery group (*p *< 0.01). Significant difference between age and post-operative pain (p = 0.011), after controlling for age and gender a significant difference in post-operative pain was still shown (*p *= 0.002).
Aydoğdu et al. [23]	Day 1 and 7	Scale of 0–4	Higher oedema in conventional osteotomy than piezosurgery on day 1 and 7 (all values *p *< 0.001)	Scale of 0–4	Higher ecchymosis in conventional osteotomy than piezosurgery on day 1 and 7 (all values *p *< 0.001)	SNOT-22, PNIF, and CCCRC did not show a statistically significant difference between the groups.
Koc et al. [24]	Day 1 and 7	Scale of 0–3	Higher oedema in conventional osteotomy than piezosurgery on day 1 (*p *< 0.001) and 7 (*p *= 0.005).	Scale of 0–3	Higher ecchymosis in conventional osteotomy than piezosurgery on day 1 and 7 (*p *< 0.001 for both).	Duration of surgery was significantly longer in the piezosurgery group (*p *= 0.002). Piezosurgery patients experienced less pain (VAS scoring) on the first day (*p *< 0.001) and were more satisfied with their results on the 1st and 7th day.
Tirelli et al. [25]	Daily for 4 days	Scale of 0–2	Higher conventional osteotomy group than piezosurgery in the 4 day follow up period (10 vs. 2, *p *< 0.05)	Scale of 0–2	Higher conventional osteotomy group than piezosurgery in the 4 day follow up period (10 vs. 2, *p *< 0.05)	Most piezosurgery patients had minimal discomfort (VAS < 3); however, 75% of the conventional osteotomy group needed analgesic (*p *< 0.05). More mucosal injury in conventional osteotomy vs piezosurgery (5 vs. 0, *p *< 0.05). Similar duration of osteotomy time (*p *= 0.11).
Ilhan et al. [26]	Day 3 and 7 Assessed by two examiners	Scale of 0-3	Higher in conventional osteotomy group than piezosurgery on day 3 (*p *= 0.002 and 0.007) and day 7 (*p *= 0.001, second examiner did not agree, *p *= 0.061). The extent of improvement was deemed similar by one examiner (*p *> 0.5), and the second examiner indicated a greater improvement in the conventional osteotomy group than the piezosurgery group (*p *= 0.017).	Scale of 0-3	Higher in conventional osteotomy group than piezosurgery on day 3 (*p *= 0.001 for both examiners), and on day 7 (*p *= 0.001 and 0.01). Extent of the improvement in both groups was similar.	N/A

A statistically significant reduction in oedema across the whole follow up-period was found in 50% (4/8) of the papers (*p *< 0.05), whilst 25% (2/8) noted an initial significant higher incidence of oedema in conventional osteotomy patients, but no difference after 7 days (*p *> 0.05). When two assessors were used, oedema had reduced on the first day of recording (day 3), but there was a disagreement on a significant difference in oedema on day 7 (*p *= 0.001, *p *= 0.061). One study believed there is no significant difference in oedema.

Three studies compared the progression of decline in eyelid oedema; similar rates of decline (*p *< 0.05), increased decline in the conventional osteotomy group (*p *= 0.017), and an initial increase in oedema in the piezosurgery group (*p *= 0.007) were found.

Regarding ecchymosis: 87.5% of the studies (7/8) identified a higher incidence of ecchymosis in conventional osteotomy patients on the first day of recording (*p *< 0.05) and a significant decrease in ecchymosis was seen at the end of the follow-up period in 75% (6/8) of these (*p *< 0.05). One paper reported no significant difference between the groups on both days.

The extent of ecchymosis decline was significantly similar across both groups of patients in 100% (3/3) of the papers (*p *< 0.05).

### Other Outcome Measurements

100% (4/4) of the studies recording post-operative pain found reduced pain in piezosurgery patients, with patients either experiencing less initial pain (*p *< 0.001, *p *< 0.05, *p *= 0.03) or reduced pain over the entire follow-up period (*p *< 0.01). Increased mucosal injury was seen in conventional osteotomy patients (*p *= 0.001 and *p *< 0.05) in 100% (2/2) of the papers. Surgery time findings varied, with a non-significant difference in surgery length (*p *= 0.11), increased time to perform piezosurgery (*p *= 0.002), and an increase in piezosurgery time (*p *< 0.001), but no significant difference in overall operation time is reported. Patient satisfaction was lower in conventional osteotomy patients. No significant difference was found in SNOT-22, PNIF and CCCRC scoring between the two groups.

## Discussion

### Summary of Main Results

This systematic review found piezosurgery patients generally had reduced oedema and ecchymosis post-surgery, with the greatest improvement in ecchymosis severity. Other secondary outcomes were reduced in piezosurgery (pain and mucosal injury). The difference in reported surgery time varied.

Taşkın et al. [19] found no significant difference in oedema between patients undergoing a conventional osteotomy vs piezosurgery.

Koçak et al. [21] and Fallahi et al. [22] found an initial higher oedema score in osteotomy patients compared to piezosurgery, with no significant difference after 7 days. Ilhan et al. [26] recorded similar findings by one examiner, although the second examiner believed there is a significant difference in oedema across the whole time period.

The remaining 4 papers (Aydoğdu et al. [23], Koc et al. [24], Tirelli et al. [25], Ghavimi et al. [20]) found a significantly higher oedema score in osteotomy patients than piezosurgery patients across the whole follow-up period.

Three papers reported on the decline in eyelid oedema. Significant declines in oedema across the time period were identified by Taşkın et al. [19] A similar extent of a reduction in oedema was reported by Ilhan et al. [26], although one examiner indicated a greater decline in oedema in the conventional osteotomy group than the piezosurgery group.

Koçak et al. [21] found a significant decline in oedema in osteotomy group over the 7 days, and in the piezosurgery group oedema worsened between days 2 and 4 and then decreased by day 7.

Taşkın et al. [19] found no significant difference in ecchymosis between patients undergoing a conventional osteotomy vs piezosurgery. The remaining 7 authors all reported a significantly higher ecchymosis score in conventional osteotomy patients compared to piezosurgery patients across the whole follow-up period.

Three papers reported on the decline in eyelid ecchymosis. Significant declines in ecchymosis across the time period were identified by Taşkın et al. [19] and Koçak et al. [21], and a similar extent of a reduction in ecchymosis was reported by Ilhan et al. [26].

### Clinical Relevance

Some studies reported additional outcome variables. Piezosurgery patients reported less pain over the whole time period according to Fallahi et al. [22] and reduced initial pain post-surgery according to Koçak et al. [21], Tirelli et al. [25] and Koc et al. [24]. Mucosal injury was significantly less common in piezosurgery according to Koçak et al. [21], and Tirelli et al. [25]. Koçak et al. [21] reported piezo surgery requiring a longer osteotomy time but no significant change in overall surgery duration, Tirelli et al. [25] reported similar surgery times, and Koc et al. [24] indicated a significantly longer surgery duration in the piezo surgery group than the conventional osteotomy group. Patient satisfaction was higher in the piezosurgery group [24]. Aydoğdu et al. [23] compared SNOT-22, PNIF, and CCCRC scoring between the two surgery groups, although a significant difference was not found.

Oedema and ecchymosis are caused by soft tissue damage during the osteotomy, often associated with angular artery injury [11]. Subsequent effects on healing and scar tissue formation can alter the aesthetic result and are thus necessary to reduce [27]. Often patients opt to rest and postpone regular activities until this subsides and is therefore important in patient recovery.

Factors affecting the degree of oedema and ecchymosis include variation in skin and nasal bone thickness. Only Ghavimi et al. [20] explicitly stated nasal bone thickness > 3mm as an exclusion criteria. An initial increased incidence of oedema and ecchymosis in patients with thicker nasal skin has been recorded, thought to increase obstruction during surgery and interfere with lymphatic drainage [28]. The relationship between nasal bone thickness and osteotome size also impacts oedema and ecchymosis [29]. Therefore, understanding how the osteotomy technique can be modified in patients with thicker nasal skin/bones could improve outcomes in these patients.

A reduction in pain, oedema, and ecchymosis have all been shown in the reports included in this review. Moreover, to patient outcomes, piezosurgery has additional technical advantages over conventional osteotomies. More accurate cuts can be made with a piezoelectric device due to the minimal force required on the piezoelectric saw [8]. Additionally, piezosurgery has shown increased precision in other surgical techniques such as bone harvesting [30]. Rabbit cadaver studies showed a reduction in bone debris in piezosurgery; excess bone debris can lead to an increased risk of post-operative infection. Lastly, diminished reductions in coagulation and tissue necrosis were also observed, potentially explaining the reduction in oedema and ecchymosis [31].

Piezosurgery, however, has its’ disadvantages. As a novel technique, inexperience of surgeons can lead to increased operation times. There were not conclusive differences in surgery time across the 8 papers, with Koc et al. [24] noting an increase in surgery time, but Koçak et al. [21] and Tirelli et al. [25] reporting no differences to overall operation time. Initially, the time to perform piezosurgery may be increased due to surgeon inexperience, however, as the learning curve progresses this is expected to reduce, alongside the controlled nature of piezosurgery decreasing operation time.

Additionally, piezosurgery presents an increased risk of thermal burns to the surgeon [9]. These both can be countered by increasing surgeon experience. As the bone is cut away with a saw, greenstick fractures can be produced instead of a complete osteotomy [7]. Lastly, the PMD device is more costly compared to conventional osteotomies in addition to buying separate tips; however, there is a minimal difference in cost to the patient. The short-term reduction in oedema and ecchymosis coupled with the improved surgery precision, pain, and reduced mucosal injury can justify the increased cost needed to procure the PMD device. The cost of the device can be offset by maximising the applications, as the PMD device can be used in bony and cartilaginous modifications to the nose such as septoplasty and modification of grafts [32].

### Limitations

Limitations of this systematic review include a minimal variation in the surgical technique performed as different approaches may lead to differing outcomes. There were inconsistencies in the ratio of male to female patients, and current literature provides limited comparisons of outcomes between them.

Nasal skin thickness was used as an exclusion criterion in one study only. Additionally, exclusion of comorbidities was more extensive in some studies than others. Peri-operatively, blood pressure was not standardised across the trials. Lastly, the follow-up period across all studies was short (< 7 days).

Future studies would need to investigate the difference in outcomes between males and females, which could lead to discoveries of optimal surgical types for each gender if necessary. A comparison between various osteotomy techniques with piezosurgery is needed as the majority included in this review use a lateral osteotomy approach, and we therefore do not know the outcomes of other conventional osteotomy approaches. The degree of dissection was not clearly stated in all papers; in the future, standardising the dissection plane will provide a more accurate comparison.

Grouping patients by comorbidity would aid in understanding the extent of interference other conditions have with prognosis. Performing surgeries with a standardised blood pressure would remove the uncertainty of its impact on oedema. Longer trials are necessary to understand the progression of oedema and ecchymosis post-surgery, as 7 days is not significant enough to understand the reduction in swelling as various techniques may have different healing rates.

In conclusion, this systematic supported the notion that piezosurgery decreases oedema and ecchymosis, pain, and mucosal injury compared to conventional osteotomies. With piezosurgery being a relatively new technique, surgery times and risk of injury to the surgeon may be increased.

In practice, piezosurgery is showing multiple benefits over conventional osteotomies in patient outcomes; increasing surgical experience of this novel technique would improve surgical issues.
